# Challenges and considerations in diagnosing the kidney disease in deteriorating graft function

**DOI:** 10.1111/j.1432-2277.2012.01516.x

**Published:** 2012-06-28

**Authors:** Henrik Ekberg, Martin E Johansson

**Affiliations:** 1 Department of Nephrology and Transplantation, Skåne University Hospital, Lund UniversityMalmö, Sweden; 2 Department of Pathology, Skåne University Hospital, Lund UniversityMalmö, Sweden

**Keywords:** calcineurin inhibitor, graft failure, nephrotoxicity, transplantation

## Abstract

Despite significant reductions in acute-rejection rates with the introduction of calcineurin inhibitor (CNI)-based immunosuppressive therapy, improvements in long-term graft survival in renal transplantation have been mixed. Improving long-term graft survival continues to present a major challenge in the management of kidney-transplant patients. CNIs are a key component of immunosuppressive therapy, and chronic CNI toxicity has been widely thought to be a major factor in late graft failure. However, recent studies examining the causes of late graft failure in detail have challenged this view, highlighting the importance of antibody-mediated rejection and other factors. In addition, the diagnosis of CNI nephrotoxicity represents a challenge to clinicians, with the potential for over-diagnosis and an inappropriate reduction in immunosuppressive therapy. When graft function is deteriorating, accurately determining the cause of the kidney disease is essential for effective long-term management of the patient. Diagnosis requires a thorough clinical investigation, and in the majority of cases a specific cause can be identified.

## Introduction

Despite significant reductions in acute-rejection rates seen with the introduction of tacrolimus, mycophenolate mofetil (MMF) and interleukin-2 receptor antibody induction in the 1990s, improvements in long-term graft survival in renal transplantation have been mixed. In Europe, data from the Collaborative Transplant Study show substantial improvements in graft survival half-life [1]. In an analysis of deceased donor kidney transplants, half-life increased from 12.5 years in 1988–1990 to 21.8 years in 2003–2005 [1]. Data from the US demonstrate more modest gains, with graft survival half-life increasing from 6.6 years in 1989 to 8.8 years in 2005 for deceased-donor kidney transplants [2]. High-risk patients showed the greatest improvement, with values for expanded criteria donors rising from 3 years in 1989 to 6.4 years in 2005, whereas low-risk patients showed little change (living donor transplant recipients: 1989, 11.4 years; 2005, 11.9 years) [2]. Reasons for the variation between the European and US findings are unclear, although the extent of possible under-reporting of death and graft loss could potentially lead to differences. Disparities may exist between the patient populations regarding accessibility to healthcare [3], and educational and socio-economic factors are also likely to have an effect. In both analyses, definition of failure included patient death with a functioning graft, but there may be other differences in statistical methodology.

Even with the gains seen in Europe, long-term graft loss is still a significant problem in renal transplantation. Improving long-term graft survival represents a major challenge in the management of kidney-transplant patients, as a wide range of factors can contribute to transplant loss [4].

Providing immunosuppressive therapy that is effective for the individual patient is an important part of transplant management. Calcineurin inhibitors (CNIs) are a key component of immunosuppressive regimens, yet chronic CNI toxicity has also been widely thought to be a major factor in late graft failure. Recent studies, however, have challenged this view. Findings from the Long-term Deterioration of Kidney Allograft Function (DeKAF) and the Genome Canada studies showed that antibody-mediated injury was a predictor of graft loss, whereas a biopsy diagnosis of ‘CNI nephrotoxicity’ was not [5–7]. Accurately determining the causes of deteriorating graft function is therefore essential to reduce the incidence of late graft failure and improve patient outcomes.

## Diagnosing the cause of deteriorating graft function

A large number of nonimmunological and immunological factors can potentially contribute to deteriorating function and graft loss following renal transplantation [8,9]. Both donor and recipient characteristics affect the risk of graft failure (Table 1); for example, donor age >60 years, a female donor and long ischaemia times are associated with increased risk. Similarly, recipient gender and the presence of comorbidities can impact on graft function. Among the immunological factors, poor human leukocyte antigen (HLA) matching, prior sensitization and inadequate immunosuppression can adversely affect outcomes.

**Table 1:** Risk factors for graft failure.

Donor factors	Recipient factors	Immunological factors
Deceased donor; donation after cardiac death Age >60 years Female gender Vascular disease or comorbidity Long ischaemia times Delayed graft function	Female gender Size mismatch Obesity Comorbidities (e.g. hypertension; hyperlipidaemia; diabetes) Proteinuria Smoking Nonadherence	Poor human leukocyte antigen matching Prior sensitization Inadequate immunosuppression

Risk-factor analyses of graft failure are after-the-fact evaluations performed in a defined cohort of patients. Although such analyses are valuable in identifying those at risk of graft failure, this is quite different from defining the cause of graft function deterioration in an individual patient. In these cases, the first, and often late, sign of a negative development is an increase in serum creatinine. The first, but sometimes insufficient, step in determining causality is to perform a graft biopsy.

### Causes of late graft failure

Transplant biopsies may be helpful in determining the potential causes of deteriorating graft function (Table 2). Early biopsy, however, is important in determining causality as the histological changes associated with chronic and severe renal dysfunction are often unspecific and uninformative in terms of useful diagnostic information. At least two biopsy cores are recommended for analysis, and serial sections are examined using a variety of stains, immunostaining and often electron microscopy. The fibrosis and sclerosing changes observed in graft biopsies from deteriorating organs can result from a variety of causes, which can typically be identified through characteristic histological changes [10,11]. Polyoma (BK) virus nephropathy [12], glomerular diseases (either *de novo* or recurrent), chronic hypertension and obstruction can all be identified from biopsies. Chronic CNI nephrotoxicity may be suggested by a number of histological lesions, although these are not specific, so diagnosis relies on elimination of other potential causes [13–15]. Chronic graft injury is associated with specific histopathological features, and C4d staining and testing for donor-specific antibodies (DSA) are important tools in helping to identify causality with chronic antibody-mediated rejection (AMR). If no clear diagnosis is apparent from the biopsy then other investigations need to be considered, such as testing for viral or bacterial infection, or ultrasound to assess arterial blood flow and exclude ureteral obstruction.

**Table 2:** Reported causes of graft failure.

Immunological	Nonimmunological
Antibody-mediated rejection T-cell-mediated rejection Nonadherence to treatment	Glomerular disease (recurrent or *de novo*) Urinary tract infection and graft pyelonephritis Polyoma (BK) virus nephropathy Calcineurin inhibitor nephrotoxicity Ureteral obstruction Vascular stenosis Thrombosis

### Definitive diagnosis of late graft failure

Determining the cause of late graft failure presents a considerable challenge, given the interactions between the factors that can affect graft function, and the difficulty of making definitive diagnoses from graft biopsies. Consequently, many cases of late graft failure are classified using the nonspecific term ‘interstitial fibrosis and tubular atrophy (IF/TA), not otherwise specified’, previously known as chronic allograft nephropathy (CAN) [11]. The recent Banff pathology consensus urged for a minimal usage of these terms as they represent a description of the histological changes observed in the biopsy rather than a specific histopathological entity and diagnosis. Widespread use of these unspecific descriptive terms may prove a hindrance to identifying and managing the real and identifiable underlying causes of graft dysfunction [11].

The slow decline in renal function coupled with absence of evidence of acute rejection has led to the view that nonimmunological factors, such as CNI nephrotoxicity, are the most important causes in late graft failure. However, given concerns that the actual causes of late kidney allograft dysfunction and failure may be being overlooked, a number of recent studies set out to examine these causes in detail (Table 3).

**Table 3:** Causes of late graft failure: a summary of recent studies.

Study	Causes of graft failure	*n* (%)
El-Zoghby *et al.* [16] *n* = 1317	*Total graft failures*	*153 (12)*
	Acute rejection	18 (12)
	Glomerular disease	56 (37)
	Medical/surgical	25 (16)
	Unknown cause	7 (5)
	Fibrosis/atrophy (IF/TA)	47 (31)
	Causes of fibrosis/atrophy	
	Polyoma virus nephropathy	11 (23)
	Immunologic (recurrent rejections)	13 (28)
	Recurrent pyelonephritis	7 (15)
	Poor allograft quality	4 (9)
	Ureteral stenosis	2 (4)
	Calcineurin inhibitor toxicity	1 (2)
	Idiopathic	9 (19)
Gourishankar *et al.* [17] *n* = 2427	*Total graft failures*	*48*
	Rejection	(38)
	Thrombosis	(17)
	Viral nephropathy	(10)
	Others	(35)
Einecke *et al.* [18] *n* = 173	*Total graft failures*	27*
	Banff classification	
	C4d+ antibody-mediated rejection	7 (26)
	T-cell-mediated rejection†	6 (22)
	Glomerulonephritis	6 (22)
	IF/TA	1 (4)
	Others	7 (26)
	Modified antibody-mediated-rejection definition‡	
	C4d+ antibody-mediated rejection	7 (27)
	C4d− antibody-mediated rejection (PRA+ with microcirculation changes)	10 (38)
	Panel-reactive antibody positive without microcirculation changes	1 (4)
	Panel-reactive antibody negative	2 (8)
	Glomerulonephritis	6 (23)
Sellarés *et al.* [19] *n* = 315	*Total graft failures*	*60*
	Acute rejection§	36 (64)¶
	Glomerulonephritis	10 (18)
	Polyoma virus nephropathy	4 (7)
	Intercurrent medical/surgical events	6 (11)
	Missing information	4 (7)

* Late graft failures (>12 months after transplantation) only.

† T-cell-mediated rejection or borderline T-cell-mediated rejection.

‡ *n* = 26 (PRA data not available for one patient).

§ Data include antibody-mediated rejection, probable antibody-mediated rejection and mixed rejection.

¶ Percentage based on *n* = 56 (causality could not be attributed in four cases because of missing clinical information).

Evaluation of over 1300 kidney-transplant recipients by El-Zoghby *et al.* showed that most cases of kidney allograft failure could be attributed to a specific cause [16]. Death with function was the most common cause of graft loss, accounting for 43% of the 330 grafts lost, whereas primary graft nonfunction accounted for 12%. Among the losses because of graft failure (*n* = 153), glomerular diseases were the most common cause (37%), whereas around 30% of cases were described as IF/TA; however, most of these could be attributed to a specific cause (Table 3). Immunological mechanisms were a common cause of graft loss among these cases, and only one case was attributed to CNI nephrotoxicity [16].

The ongoing, observational DeKAF study, conducted at seven centres in the USA and Canada, aims to identify and characterize the causes of late kidney allograft dysfunction and failure using two different patient cohorts [5,6,17]. The cross-sectional cohort includes patients transplanted prior to October 2005 who developed new-onset late graft dysfunction, and provides information on ‘the troubled kidney’, irrespective of the time from transplantation. The prospective cohort includes patients transplanted after 1 October 2005 who were enrolled at the time of transplantation, and provides information on all kidney-transplant recipients irrespective of outcome. In this prospective cohort, there were 103 graft losses as of February 2009, with 55 of these because of death with a functioning graft. Among the remaining 48 cases, rejection, thrombosis and viral nephropathy were common causes of graft loss (Table 3).

**Figure 1:**
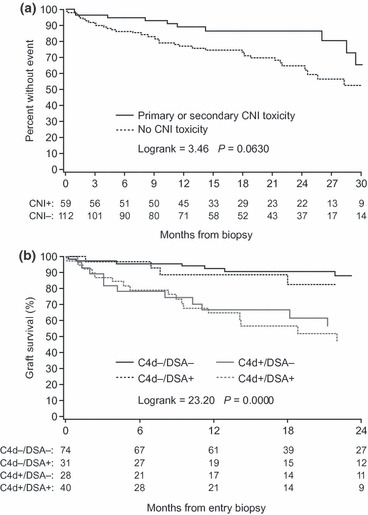
Kaplan–Meier estimates of the impact of (a) diagnosis of CNI toxicity and (b) presence or absence of C4d and DSA on graft survival in the DeKAF study [5]. Reprinted from: Gaston *et al.* [5].

Data from the cross-sectional cohort showed that patients with new-onset late allograft dysfunction who were diagnosed with ‘CNI toxicity’ had slightly lower rates of graft failure than those without this diagnosis [5]. Kaplan–Meier analysis showed improved graft survival following biopsy for patients with ‘CNI toxicity’ compared with those with no ‘CNI toxicity’, based on the local pathologist’s diagnosis at enrolment (Fig. 1a). Similarly, postbiopsy graft survival did not differ significantly between patients with or without a local pathologist’s diagnosis of CAN, and the postbiopsy slope of 1/creatinine versus time was also similar in the two groups [17]. By contrast, the presence of inflammatory cell infiltrates in regions of fibrosis and atrophy in the transplant biopsy was strongly associated with graft failure – although these findings did not qualify for a Banff diagnosis of rejection [6]. Even after adjusting for renal function at biopsy, or the extent of interstitial fibrosis or tubular atrophy in the biopsy, inflammation in these regions showed a strong association with graft failure.

Evidence from the DeKAF study demonstrates the importance of antibody-mediated injury in late graft failure [5]. In the study, 57% of the patients analysed were positive for C4d staining and/or DSA. Both C4d staining and the presence of DSA were associated with significant increases in the risk of postbiopsy graft failure. Analysing the patients in four groups according to their C4d/DSA status showed that C4d+/DSA+ patients had the highest risk of graft failure, whereas the risk was quite low in C4d−/DSA− patients (Fig. 1b).

Antibody-mediated microcirculatory injury was found to be a major cause of late graft failure in a study evaluating unselected kidney-transplant biopsies for clinical indication from 173 patients (Table 3) [18]. Almost all graft failures occurred in grafts biopsied 1 year after transplantation, and analysis showed that microcirculatory changes (particularly glomerulitis) and scarring were associated with late graft loss. C4d staining was not a good predictor of graft loss. However, AMR was most frequently associated with graft loss when it was redefined to include other characteristic features such as the presence of HLA antibody and microcirculatory changes, irrespective of C4d status. This suggests that many cases of antibody-mediated acute rejection may be misclassified using diagnostic criteria that only rely on C4d+ staining. Interestingly, biopsy changes characteristic of T-cell-mediated rejection and CNI toxicity were not associated with an increased risk of graft loss [18].

Antibody-mediated rejection was also found to have a dominant role in graft failure in a prospective study of over 300 renal transplant recipients enrolled at the time of clinical-indication biopsy [19]. This study aimed to determine the cause of all graft failures, based on biopsy diagnosis, HLA antibody status and clinical information, and showed that rejection (64%) and glomerulonephritis (18%) were the main causes of graft failure (Table 3). No graft losses were attributed to CNI toxicity or unexplained fibrosis. Importantly, nonadherence to antirejection therapy was more prevalent among patients who progressed to graft failure (32%) than those who did not (3%). Among patients experiencing failure because of acute rejection, 47% were nonadherent, underlining the importance of treatment adherence to delay late graft failure [19]. In addition, both interstitial inflammation and severe tubulitis were associated with poorer graft survival in late biopsies [7]. In multivariate analyses, only the presence of progressive diseases was significantly associated with graft loss, suggesting that inflammation occurs as part of a secondary injury–repair mechanism in response to progressive diseases (for example AMR and glomerulonephritis), rather than driving renal deterioration independent of disease.

### Is CNI nephrotoxicity over-diagnosed?

Given the evidence for the role of immunological factors and specific diseases in late graft loss, this raises the question of whether CNIs really are the main cause of progressive structural damage to the allograft, and whether the case for their nephrotoxicity has been overstated.

Acute CNI nephrotoxicity is well documented, with high blood levels of CNIs associated with decreases in renal function and histological changes to the kidney. These changes are typically reversible with CNI withdrawal [14,20]. Vasoconstriction of the afferent arterioles and direct effects of CNIs on the tubular epithelium are thought to underlie the mechanisms of acute CNI nephrotoxicity [14]. The evidence for chronic CNI nephrotoxicity is less clear cut, with much of the data coming from extra-renal transplant studies [14,20]. However, the extent to which other causes contribute to late graft dysfunction in these studies is unclear [21].

The main histological lesions classically associated with CNI toxicity include arteriolar hyalinosis, striped fibrosis, glomerulosclerosis, tubular atrophy and microcalcifications (Table 4) [14]. All histological changes may have other causes, and thus diagnosis is based on the combined occurrence of several of the criteria and the elimination of other potential causes, rendering CNI toxicity a diagnosis of exclusion [13]. As a result, the criteria used for diagnosis can differ between institutions and studies, making comparisons difficult.

**Table 4:** Histological lesions associated with chronic CNI toxicity and differential diagnosis.

Chronic CNI toxicity	Differential diagnosis	Comment
Interstitial fibrosis and tubular atrophy (typically striped)	Pre-existing donor injury, ageing, ischaemia-reperfusion injury, tubulo-interstitial rejection, infection (e.g. UTI, polyoma virus, CMV), chronic ischaemia (e.g. renal artery stenosis, size discrepancy in paediatric transplantation), chronic post-renal obstruction, diabetes mellitus	Today regarded as nonspecific and of nondiagnostic value. Reflects loss of nephrons regardless of cause
Arteriolar medial hyalinosis	Pre-existing donor injury, ageing, diabetes mellitus, hypertension (in these cases more subendothelial deposition)	–
Glomerular capsular fibrosis	Glomerular ischaemia (e.g. renal artery stenosis, chronic arteriolar vasoconstriction or arteriolar hyalinosis) and other causes of atubular glomeruli (i.e. causes of tubular atrophy)	Unspecific sign of nephron injury
Global glomerulosclerosis	Pre-existing donor injury, ageing, chronic glomerular ischaemia (e.g. renal artery stenosis, arteriolar vasoconstriction or hyalinosis), recurrent primary disease, *de novo* glomerular disease, hypertension secondary to tubular atrophy in a late stage	Unspecific sign of renal injury and with no diagnostic value except for calculating degree of glomerular loss
Focal segmental glomerulosclerosis	Recurrent primary disease, donor–recipient size discrepancy with hyperfiltration injury, FSGS secondary to other causes of glomerulosclerosis	–
Juxtaglomerular apparatus hyperplasia	Not well established, but likely other causes of hyperreninaemia (e.g. transplant renal artery stenosis)	Uncommon and of uncertain diagnostic value
Tubular microcalcifications	Pre-existing donor injury, ischaemic tubular injury and acute tubular necrosis, bone and mineral metabolism imbalance, proteinuria	Unspecific sign of renal-tubular injury

CMV, cytomegalovirus; CNI, calcineurin inhibitor; FSGS, focal segmental glomerulosclerosis; UTI, urinary tract infection.

Adapted from: Naesens *et al.* [14].

The extent of CNI toxicity observed in biopsy specimens varies considerably between studies. A study by Nankivell *et al.* in patients receiving kidney–pancreas transplants suggested that evidence of CNI toxicity was present in almost all biopsy specimens at 10 years post-transplant [22]. However, this study lacked a control group to assess the effects of other potential factors on renal function; rejection, infections, other nephrotoxic drugs, comorbidities and the ageing process can all adversely affect renal function. Furthermore, long-term outcomes in the study were good, despite the histological changes, with death-censored kidney graft survival rates of 95% at 10 years, suggesting that CNI toxicity was not associated with late graft failure. The relatively limited number of late biopsies, evidence for the occurrence of ongoing subclinical rejection in the patient population, and the early appearance of fibrosis with little subsequent progression suggest that other factors could have played a role in the development of histological lesions [21].

Other studies with CNI therapy suggest that histological changes associated with CNI toxicity are less common; biopsy studies have reported rates of CNI toxicity as low as 4%, although their shorter duration and differences in diagnostic criteria make comparisons difficult [23]. Evidence also suggests that histological changes show little progression over time and have little impact on graft function. In a recent study, evaluation of biopsies taken 1 and 5 years after kidney transplantation showed that the prevalence of chronic histological changes was low, and few patients with biopsies at both time points showed progression of interstitial fibrosis [24]. Furthermore, the prevalence of arteriolar hyalinosis at 5 years was similar in CNI- (tacrolimus-) treated and CNI-free (sirolimus-treated) patients. The number of sirolimus-treated patients in the study was low.

In the DeKAF study, analysis showed that almost all biopsies had IF/TA, hence would meet the nonspecific criteria for IF/TA [25]. However, cluster analysis based on central Banff scores identified distinct subgroups of patients with differing characteristics and outcomes. Patients with mild fibrosis and atrophy but no inflammation had good outcomes, whereas the combination of fibrosis, atrophy and significant inflammation was associated with poorer outcomes [25]. Similarly, in another analysis of biopsies at 1 year following kidney transplantation, patients with fibrosis alone showed similar graft survival compared with those with normal biopsies, whereas the addition of inflammation was associated with reduced graft survival [26]. This suggests that biopsies showing fibrosis/atrophy alone, but no evidence of active inflammation (or recurrent disease), were not associated with subsequent deterioration in renal function.

The difficulties in diagnosing CNI toxicity were apparent in a retrospective analysis comparing protocol biopsies (3 months, 2 years and 10 years post-transplant) from CNI- (ciclosporin-) treated and CNI-free kidney-transplant patients [15]. Although histological changes associated with CNI toxicity progressed over time, and were more frequent in CNI-treated than CNI-free patients, particularly at the later assessments, lesions were observed in patients who had not received CNI therapy. Arteriolar hyalinosis was observed in over 90% of patients receiving CNIs at 10 years post-transplant, but also occurred in 65% of CNI-free patients. Muscular arteriolar hyalinosis, considered even more specific for CNI toxicity, was present in 28% of CNI-free versus 68% of CNI-treated patients. An editorial commentary concluded that ‘there is no such thing as a specific histological diagnosis of CNI nephrotoxicity in an individual patient’ and also suggested the possibility of undiagnosed AMR in the CNI group, which could have adversely affected outcomes [13].

### Chronic CNI toxicity and extra-renal transplants

Chronic renal failure is a serious problem in nonrenal transplant recipients, raising concerns about the potential effects of CNI therapy on renal function in these patients. An analysis of over 69 000 nonrenal solid organ transplants showed that 16.5% of patients developed chronic renal failure during follow-up (median 36 months), with the 5-year cumulative incidence ranging from 7% to 21%, depending on the type of transplant [27]. Multivariate analyses showed that a range of factors including age, pretransplant glomerular filtration rate (GFR) and the presence of hypertension, diabetes or hepatitis C infection was associated with an increased risk of chronic renal failure. Use of CNI-based therapy at the initial hospitalization for transplantation was also associated with increased risk, and in liver transplant recipients the risk of renal failure was greater with initial ciclosporin versus tacrolimus treatment. Interestingly, renal failure was more common in liver than heart transplant recipients [27], even though heart transplant patients are typically treated with higher CNI concentrations. This analysis underlines the multifactorial nature of renal failure in nonrenal organ transplants, which is unsurprising given the potential for pre-existing renal damage in these patients. Prolonged renal vasoconstriction is common in heart and liver failure, for example, and many patients have diabetic nephropathy, hypertension or other vascular disease, highlighting the importance of detecting and managing renal injury in these patients.

In a retrospective analysis of renal biopsies in 101 patients following nonrenal transplantation, histological changes characteristic of hypertension, CNI toxicity, primary glomerular disease and thrombotic microangiopathy were observed [28]. Other studies have shown that a range of factors can affect renal function in nonrenal transplant recipients, but have observed little evidence of CNI toxicity. A study in 81 patients who developed impaired renal function following liver transplantation found glomerular abnormalities in all biopsies [29]. The pathology was suggestive of diabetic nephropathy and hypertensive change, although specific glomerular disease processes were also present; however, little CNI toxicity was observed [29]. Similarly, renal biopsies from 18 heart transplant recipients with renal failure showed a diverse pattern of histological changes, with only one patient showing evidence of CNI toxicity, suggesting that the pathologic basis for chronic kidney disease after heart transplantation is complex and varied [30].

### Clinical implications of over-diagnosis of CNI nephrotoxicity

Over-diagnosis of CNI nephrotoxicity could lead to an inappropriate reduction in immunosuppressive therapy, putting the patient at risk of increased immunological activity [21]. This has the potential to lead to a vicious circle of deteriorating graft function, graft biopsy misinterpretation of ‘CNI toxicity’ and a reduction in CNI dosing and therefore in immunosuppressive efficacy. Reduced immunosuppressive efficacy would lead to further antibody-mediated injury and hence to a worsening of graft function and graft failure. As discussed above, AMR is an important cause of late graft failure [18,19,31]; ensuring adequate immunosuppression is therefore a key aspect of patient management.

Evidence from clinical trials shows that CNI-based regimens provide similar renal function and equivalent or superior graft survival to alternative regimens. The CAESAR study showed that patients receiving a ciclosporin withdrawal regimen showed similar renal function to low- or standard-dose ciclosporin therapy, but had a higher incidence of biopsy-confirmed acute rejection (BCAR) [32]. In the recently published ORION study, sirolimus-based regimens were not associated with improved outcomes compared with tacrolimus-based treatment in renal transplant patients, and higher than expected levels of BCAR were observed in the sirolimus plus MMF arm [33]. A study with a sotrastaurin plus mycophenolic acid (MPA)-based regimen showed improved renal function compared with tacrolimus plus MPA-based therapy, but higher rates of BCAR, leading to early study termination [34]. In the ELITE-Symphony study, graft function and survival were superior with low-dose tacrolimus (plus MMF and corticosteroid) therapy compared with either sirolimus, or low- or standard-dose ciclosporin treatments (all with MMF and corticosteroids) [35]. The BENEFIT and BENEFIT-EXT studies showed improved renal function with belatacept-based versus ciclosporin-based treatment, although graft survival was similar in the two groups and BCAR and post-transplant lymphoproliferative disorder (PTLD) were increased with belatacept therapy [36,37]. Furthermore, the improved outcomes with CNI-based therapy have occurred despite the worsening risk profile for both donors and recipients, including ageing of the donor and recipient populations and the increased use of expanded criteria donors.

Evidence shows that under-immunosuppression is associated with increased injury and graft loss. Some studies have shown that reduced immunosuppression therapy is associated with increased IF/TA progression [38], while a study of late biopsies (>1 year post-transplantation) from kidney-transplant recipients showed that patients who were noncompliant with CNI therapy had more inflammatory changes, more interstitial fibrosis and similar levels of arteriolar hyalinosis than compliant patients [39]. An observational study in kidney-transplant patients with good graft function showed that reduction or withdrawal of immunosuppression therapy (ciclosporin, tacrolimus or MMF) during the second year post-transplant was associated with a significant risk of graft loss [40]. As discussed above, a recent prospective study showed an increased prevalence of nonadherence among renal transplant recipients who progressed to graft failure [19].

## Managing graft deterioration: practical considerations

Preventative measures aimed at minimizing the risk of renal dysfunction are an important aspect of patient management following kidney transplantation. Hence, the impact of comorbidities such as diabetes, hypertension and hyperlipidaemia on graft function and overall patient health needs to be considered. For example, hypertension is widespread in kidney-transplant recipients, and treatment with calcium channel blockers has been shown to be well tolerated, reduce graft loss and improve GFR [41]. Other measures to reduce cardiovascular risk, such as managing hyperlipidaemia and promoting a healthy lifestyle, are also important, given the high rates of cardiovascular events observed in transplant recipients.

When graft function is deteriorating, accurately determining the cause of the kidney disease is essential for the effective management of the patient. Diagnosis requires a thorough clinical investigation, with nothing taken for granted; as discussed above, a specific cause can be identified in the majority of cases, based on a combination of biopsy diagnosis, antibody testing and clinical information.

**Figure 2:**
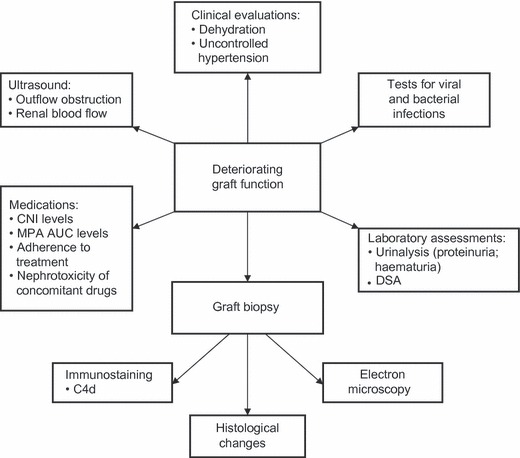
Overview of investigations to determine the cause of kidney disease in patients with deteriorating graft function. AUC, area under the curve; DSA, donor-specific antibodies; MPA, mycophenolic acid.

Investigations should include clinical assessments for dehydration, uncontrolled hypertension and infection, which can all affect renal function, whereas ultrasound should be used to assess outflow obstruction and renal circulation (Fig. 2). Proteinuria or haematuria on urinalysis provides evidence of glomerulonephritis or transplant glomerulopathy, which can be further evaluated on biopsy. CNI blood concentrations should be evaluated to ensure they are within the therapeutic window, and concomitant medications should also be considered for potential nephrotoxic effects. Tests for viral (for example, cytomegalovirus and BK virus) and bacterial infections should also be performed as these can be associated with deteriorating graft function. Screening techniques, diagnostic procedures and various therapeutic management options for BK virus, including the reduction in immunosuppressive drugs to contain infection, have recently been reviewed [12].

Biopsy assessments are a key part of the diagnostic process, with characteristic histological changes allowing the identification of a range of specific causes of graft loss. Studies show that in most cases, a specific cause of late graft failure can be identified [16,19]. Diagnosis of CNI nephrotoxicity is reliant on the elimination of other causes and does not predict graft loss, hence the importance of evaluating other potential factors in these cases. By contrast, antibody-mediated injury is an important predictor of graft loss, making C4d staining and DSA testing a key part of the evaluation process. However, C4d staining is not always reliable and it has been suggested that findings of microvascular damage in the biopsy [42] and proteinuria [43] should be considered more valuable predictors of graft loss, in combination with DSA testing.

Once a diagnosis has been made, the treatment approach should be individualized for each patient. For those with evidence of chronic AMR from biopsy or the presence of DSA, for example, immunosuppression should be increased, either by raising the dose of tacrolimus or MMF therapy or adding an mTOR (mammalian target of rapamycin) inhibitor, such as everolimus. Specific measures to reduce DSA levels may also be considered, such as the administration of bortezomib or eculizumab, although these treatment modalities should still be regarded as largely experimental [44–47]. Assessing and promoting treatment adherence may also prove beneficial in these patients, as nonadherence is frequently associated with late rejection.

## Funding

Editorial services of iS Health involved in the preparation of this manuscript were supported by Astellas Pharma Europe Ltd. No honoraria were received by the authors for the preparation of the manuscript.
